# Plant chemical variation mediates soil bacterial community composition

**DOI:** 10.1038/s41598-023-32935-4

**Published:** 2023-04-13

**Authors:** Robert W. Buchkowski, Klára Benedek, János Bálint, Attila Molnár, Tamás Felföldi, Csaba Fazakas, Oswald J. Schmitz, Adalbert Balog

**Affiliations:** 1 Department of Biology, University of Western Ontario, 1151 Richmond Street, London, ON N6A 5B7 Canada; 2 Department of Horticulture, Faculty of Technical and Human Science, Sapientia Hungarian University of Transylvania, Sighisoara Str. 1C, Targu Mures, Romania; 3 Department of Biology and Chemistry, Ferenc Rákóczi II Transcarpathian Hungarian College for Higher Education, 6, Berehove, Zakarpattia Oblast, 990201 Ukraine; 4 Department of Biology, Hungarian University of Agriculture and Live Science, Páter Károly Str. 1, Gödöllő, 2100 Hungary; 5 Department of Microbiology, Eötvös Loránd University, Pázmány Péter Stny. 1/C, Budapest, 1117 Hungary; 6 School of the Environment, Yale University, 370 Prospect Str., New-Haven, CT USA; 7 Present Address: Atlantic Forestry Centre, Natural Resources Canada, 1350 Regent Street, Fredericton, NB E3C 2G6 Canada

**Keywords:** Ecology, Evolution, Microbiology

## Abstract

An important challenge in the study of ecosystem function is resolving how plant antiherbivore chemical defence expression may influence plant-associated microbes, and nutrient release. We report on a factorial experiment that explores a mechanism underlying this interplay using individuals of the perennial plant Tansy that vary genotypically in the chemical content of their antiherbivore defenses (chemotypes). We assessed to what extent soil and its associated microbial community versus chemotype-specific litter determined the composition of the soil microbial community. Microbial diversity profiles revealed sporadic effects of chemotype litter and soil combinations. Soil source and litter type both explained the microbial communities decomposing the litter with soil source having a more important effect. Some microbial taxa are related to particular chemotypes, and thus intra-specific chemical variation of a single plant chemotype can shape the litter microbial community. But we found that ultimately the effect of fresh litter inputs from a chemotype appeared to act secondary as a filter on the composition of the microbial community, with the primary factor being the existing microbial community in the soil.

## Introduction

Understanding what controls the structure and function of terrestrial ecosystems has been greatly enhanced by considering aboveground (plant-based) and belowground (detritus-based) food chains as coupled systems^1^. This conception has given rise to the appreciation that variation in plant functional traits (e.g., nutrient content and anti-herbivore defense expression) can determine variation in the community composition of different trophic compartments (i.e., microbial decomposers, herbivores, carnivores) within ecosystems^2–6^. Compounding this complexity is the growing realization that intraspecific variation in plant functional traits can explain as much variation in food web structure and ecosystem functioning as interspecific plant trait variation^7–12^. But understanding the community- and ecosystem-wide consequences of intraspecific variation in plant trait expression remains rudimentary^7,13,14^; especially how soil bacterial communities and their functioning might respond to variation in plant traits^15^.

We report here on an experiment aimed at understanding how intraspecific variation in the nature and concentration of plant volatile chemicals that ward off insect herbivory affect soil microbial communities and their decomposition of plant litter containing volatile chemicals. The study is motivated by previous evidence that interspecific variation in plant chemical defense composition (aka plant chemotype) can influence the trophic structure of food-webs^16–19^. Our previous work, in particular, demonstrated that plant chemotype can determine both arthropod and soil microbial communities^20^. This study complements that work by resolving how plant chemotype can alter soil microbial community composition. We test whether soil microbial community composition is shaped most by the original plant chemotype with which the microbes are naturally associated or by differences in litter inputs from alternative chemotypes using chemotypes of the perennial herb Tansy (*Tanacetum vulgare*) as our system of study.

Our research combined the use of next-generation DNA sequencing (16S rRNA gene amplicon sequencing) to assess soil microbial community composition with a litter decomposition experiment to address the following questions: (1) Does a soil microbial community associated with a particular plant chemotype have a different ability to decompose litter from its own chemotype vs litter from another chemotype? (2) Does soil microbial diversity change when subjected to its own chemotype’s litter vs. another chemotyope’s litter?

## Methods

### Study system

Tansy (*T. vulgare*) is a perennial plant originating in Europe and Asia^21^. Large populations can be found in disturbed, well-drained, nutrient poor soils^22^, where it often forms isolated patches. It also frequently occurs alongside river valleys, railway tracks and on abandoned lands. Tansy genotypes can be classified according to their volatile chemical content (chemotypes): most frequent are β-thujon, camphor, and borneol^23^. Breeding experiments with these chemotypes using molecular markers have confirmed that the volatile chemical content of a particular Tansy plant is determined genetically^21,22^.

Tansy chemotypes determine their associated arthropod communities that include three specialised aphid species (*Macrosiphoniella tanacetaria* (Kaltenbach), *Metopeurum fuscoviride* Stroyan and *Uroleucon tanaceti* L.) and many predators specialised on Tansy aphids, the most important being the 7-spotted ladybird beetle (*Coccinella septempunctata*), the generalist nursery web spider (*Pisaura mirabilis*) and the minute pirate bug (*Orius* spp.)^24^. Together, these properties of Tansy make an ideal model system for studying effects of intraspecific plant variation on ecosystem functions.

**Figure 1:**
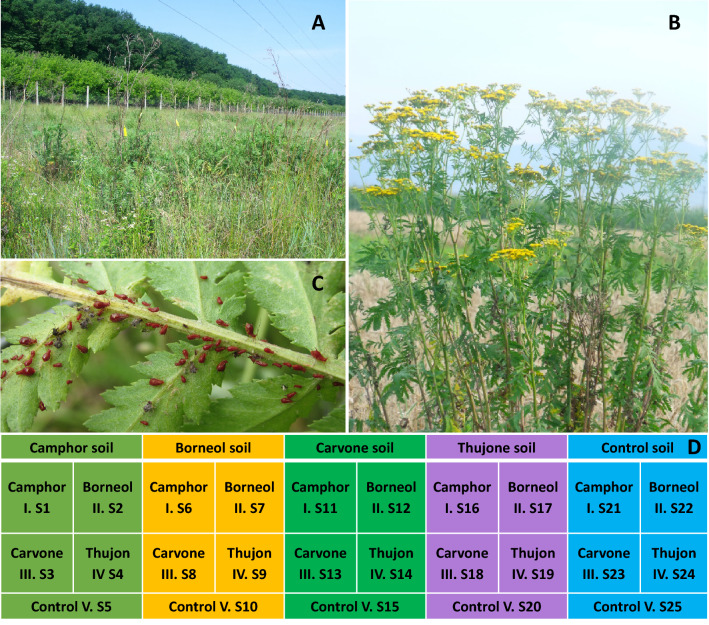
Tansy field (**A**), Tansy plant (**B**), *U. tanaceti* aphids on tansy leaves (**C**) (Photos made by Adalbert Balog), litter decomposition and soil bacterial community assay design (**D**). Numbers represents sample orders, which were used consequently in labelling samples for genetic analyses. The first four treatments representing the main chemotype soils (I, II, III, IV), the control with no tansy (V) soil were used in boxes, within each, litter additions from the different chemotypes (I-IV) or non-Tansy plants (V) were placed. Marks as S1…S25 represents particular chemotype samples in particular chemotype soil (i.e. III.S3 means Carvone litter in Camphor soil, versus III S8 Carvone litter in Borneol soil).

The experiment reported here used individuals drawn from Tansy populations that belong to different genetic types with different chemical defense profiles (chemotypes)^20^. These chemotypes were identified in previous work which surveyed and evaluated the chemical composition and genotypes of 100 tansy plants from populations along a 120 km transect in Transylvania, Central Europe^20^. That previous survey revealed that chemotypes where comprised of different compositions of four key volatile chemicals: (1) Camphor (2) Borneol (3) Carvone, (4) β-Thujon (see^20^ for details). We used soil and litter associated with hybrid chemotypes that were comprised of a mixture of 40% or more of a dominant volatile chemical and 20% or less of the other volatiles. For example, a hybrid with 40% or more Camphor comprised the Camphor treatment, a hybrid with 40% β-Thujon comprised the Thujon treatment, etc. (Fig. 1). When possible, we used litter and soils from multiple individual plants of each chemotype taken from points along the 120 km transect. We obtained soils and litter associated with Camphor, Borneol and Thujon hybrids (n = 3 plants for each hybrid chemotype), and Carvone hybrid (n = 1 plant).

We collected soils associated with the individual plants by extracting soil from a 50 cm diameter area around each plant to a 15 cm depth. This soil horizon contained 3.26% humus, a mobile potassium content of 408 ppm and nitrogen which varied between 0.143% and 0.101%. The base saturation of the upper layer was 77.85%, and the pH (H2O) 6.38^20^. We collected aboveground biomass of each individual plant by clipping them at the soil surface.

### Litter decomposition experiment

The litter decomposition experiment evaluated how soil and litter from each chemotype shaped the soil microbial community. We further evaluated whether transplanting litter from a chemotype to soils associated with another chemotype influenced the microbial community. We deployed a factorial design, crossing soil and litter sourced from each of the four hybrid chemotypes plus the control (Fig. 1).

We created treatment soils (Fig. 1) by bulking and homogenizing soil from the replicate hybrids plants for a chemotype treatment. We also collected and homogenized leaf material from each of the treatment chemotypes for the decomposition assay. We further created a control by collecting and homogenizing soil and plant material from field locations covered in monocots without tansy plants. Thirty kilograms of soil from each hybrid chemotype or control were filled into five individual 40 × 40 × 30 cm boxes per chemotype (Fig. 1).

We put a homogenized mixture of 33 g of litter and 66 g of soil from each chemotype or control into individual standard 0.2 mm mesh litterbags^25^. We added the soil to each litter bag that was from the same chemotype as the litter soil. We created 5 replicate litterbags for each chemotype or control for each soil treatment (n = 125 litter bags with n = 25 litter bags per each of the 5 soil treatments or control) At the end of November 2020, we buried the five replicate litter bags for each litter-soil treatment combination 10 cm below the soil surface within each box (Fig. 1).

All boxes were kept outdoors under natural conditions until the end of May 2021. Litterbags were then collected from each chemotype box and samples were placed into sterile tubes and stored at − 70 °C until subject to DNA analyses.

Total genomic DNA was extracted with the DNeasy PowerSoil Pro Kit (Qiagen) from the mixture of litter and soil remaining in each buried litter bag in May 2021. Then, the V3-V4 region of the 16S rRNA gene was amplified with Bacteria-specific PCR using the following primers: B341F (5′-CCT ACG GGN GGC WGC AG-3′^26^; and 805NR (5′-GAC TAC NVG GGT ATC TAA TCC-3′^27^. DNA sequencing was conducted by the Genomics Core Facility RTSF of the Michigan State University (USA) on a standard MiSeq v2 flow cell (Illumina) in a 2 × 250 bp paired end format using a v2, 500 cycle MiSeq reagent cartridge. Sequences analysis was performed with mothur v1.44.3^28^, while read alignment and taxonomic assignment were carried out using the ARB-SILVA SSU Ref NR 138 reference database^29^ applying operational taxonomic units (OTUs) at a traditional 97% cutoff. A total of 852,130 high-quality reads were obtained in this project, an average of 34,085 read/sample.

### Data analyses

Microbial community data were rarefied to 19,000 reads per sample before we created an average distance matrix for analysis using 100 random draws from each of our sequenced communities (n = 25).

First, the 13 dominant bacterial phyla and genera were compared between plant chemotypes and the control; here, only proportional differences of bacterial distributions were presented between samples using microbial sequences data. Then, we produced diversity profiles of the entire set of bacteria genera (i.e., all OTUs) to examine differences in the community diversity in different soil and litter combinations. Next, we used non-Metric Multidimensional Scaling (NMDS) to compare the composition of bacterial phyla and genera and tansy chemotype. Groupings were based on relative proportions of different chemical volatiles in each Tansy plant. Finally, we tested for a significant effect of soil type and litter type on the bacteria community using the multivariate analysis of variance (vegan::adonis2). Analyses were run in R Studio v0.97.314 using R v3.0.1^30^ (R Core Team 2013).

### Permit statement

Experimental research and field studies on tansy, including the collection of plant material, complied with institutional, national, and international guidelines and legislation. Permissions were not required for *Tanacetum vulgare* collections because tansy is a wild weed with moderate expansion in Transylvania, included between plants that has to be controlled with plant protection methods. Voucher specimens were not deposited as only leaves and stems were collected for analyses, entire specimens were not collected.

## Results

**Figure 2:**
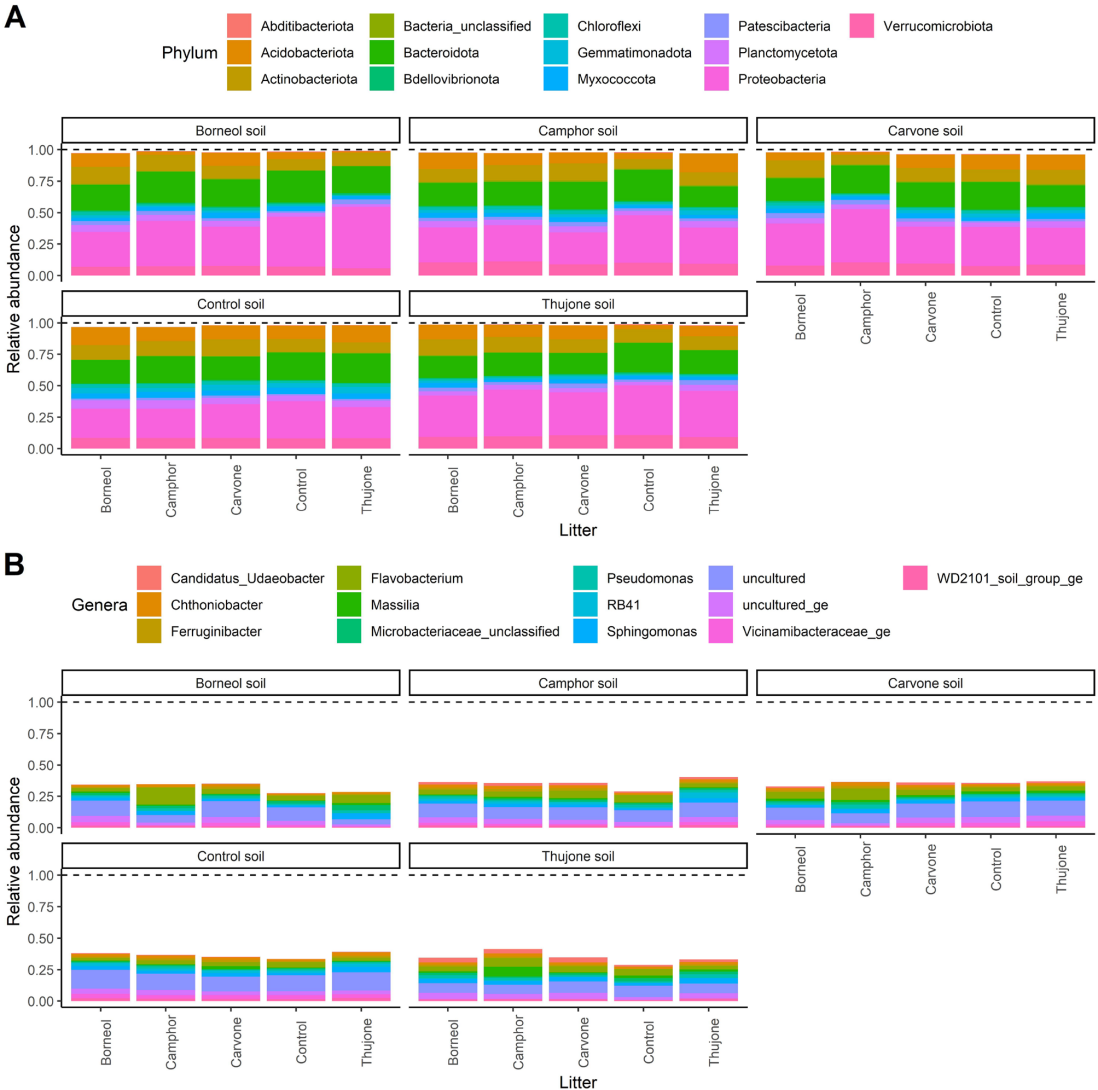
The 13 most abundant bacterial phyla (**A**) and genera (**B**) under different tansy chemotypes with own soil and with other chemotypes soil and control (no tansy). Relative abundance does not sum to 1 to account for reads that did not belong to the 13 most abundant taxa.

Bacterial communities in the litter bags from different chemotypes had a different composition of phyla (Fig. 2A) and genera (Fig. 2B) across our treatments, with changes in the abundance of phyla less pronounced than those in genera.

**Figure 3:**
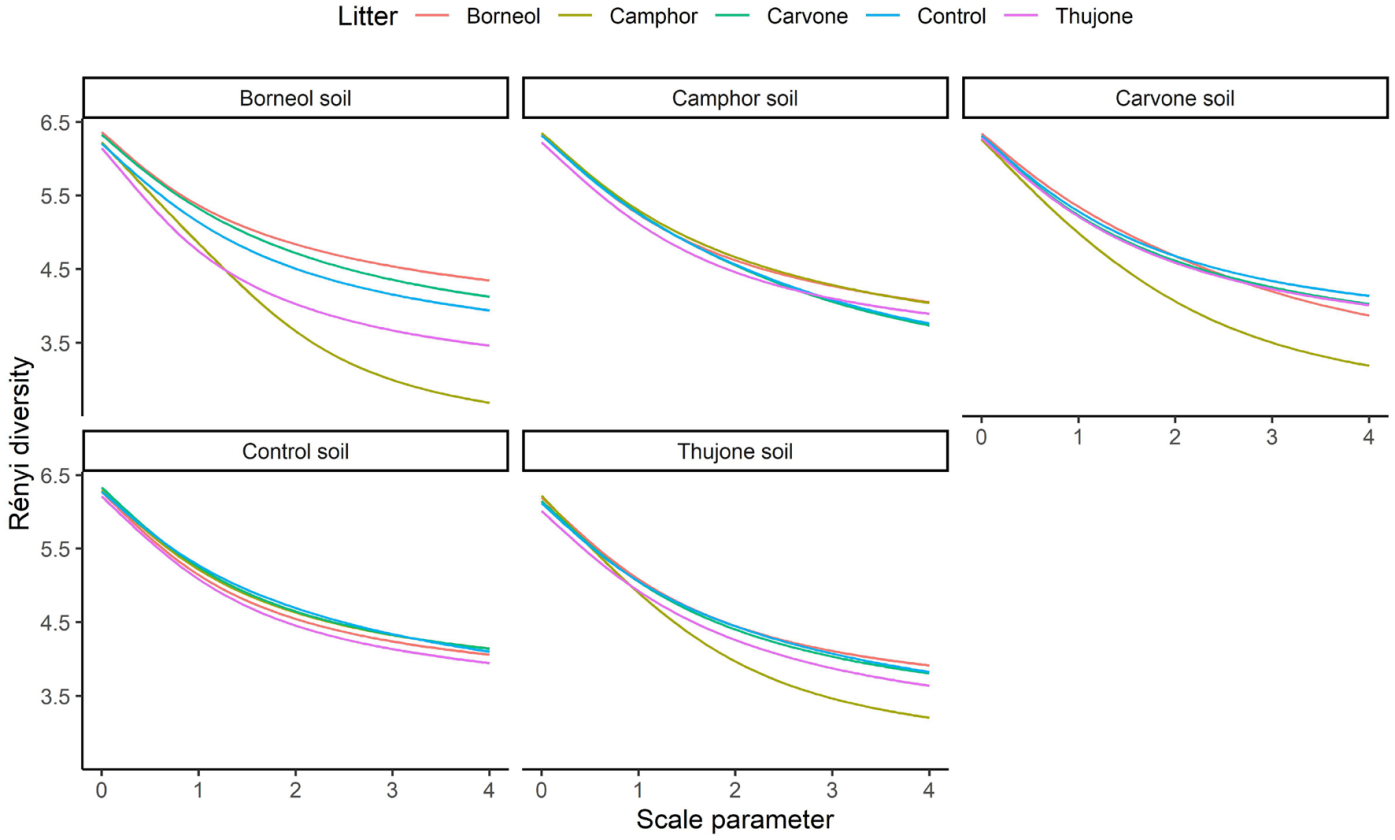
Diversity orders according to alpha diversity profiles of bacterial communities under different tansy chemotypes with own soil and with other chemotypes soil and control (no tansy).

Diversity profiles of bacteria genera revealed that soil and litter combinations had similar species richness (Fig. 3). Litter from Camphor plants decomposed in soil taken from underneath Carvone, Thujone, and Borneol plants, but not the controls, had fewer rare species as indicated by lower diversity values as the scale parameter increased (Fig. 3). Borneol soil had the largest effect on community evenness (i.e., high scale parameter) with the most diversity retained by litter from Borneol plants when it was decomposed in the soil from beneath Borneol plants. In fact, Borneol litter decomposed in soil from beneath Borneol plants was the only combination where diversity was unambiguously different—higher in this case—than other treatments (Fig. 3).

**Figure 4:**
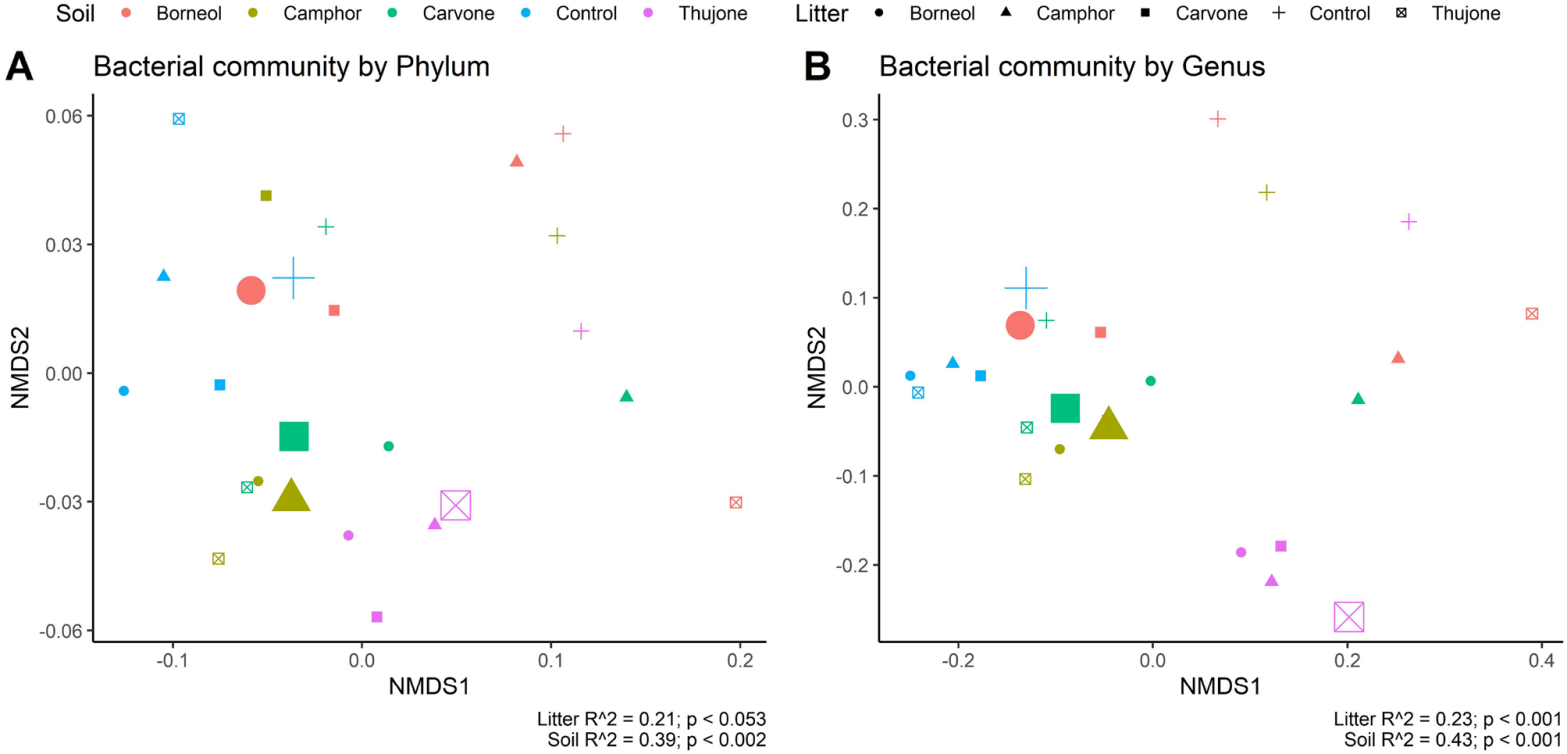
Soil and bacterial community interaction at the Phylum (**A**) and at the Genus (**B**) level. Non-Metric Multidimensional Scaling (NMDS) using microbial sequencing data were applied to reveal relational patterns among bacterial phyla and genera and tansy chemotype. The large points show where soil and litter came from the same chemotype or control site.

Both the source litter and the soil in which it was buried had a significant influence on the composition of the bacterial community. Overall, the soil in which that litter was decomposed had a strong effect on the composition of the bacterial community than by the type of litter being decomposed. This was true for both bacterial phyla and genera (Fig. 4A,B).

## Discussion

Our experiment revealed significant variation in the bacteria communities decomposing litter from different Tansey chemotypes in a common garden experiment. The variation was driven by both soil and litter sources, indicating that community assembly was significantly affected by both processes. Yet, the soil source played a dominant role, explaining twice the variation in community composition as did litter type.

Relative changes in the microbial community across chemotypes could indicate differences in function, but our data do not support this interpretation. For example, we have already demonstrated that plant and soil nitrogen increase from Thujone to Borneol to Camphor plots in the field^20^. Here, we found microorganisms, such as *Pseudomonas*, *Massilia*, and *Sphingomonas*, that have been described as important genera for litter degradation and mineralization. Their role in litter early decomposition has been demonstrated^31^. Yet, their relative abundance, individually or in total, rank sporadically across soil and litter combinations, suggesting a limited link between relative abundance and functional outcomes in the field.

So, the microbial community decomposing litter varied by both the soil source and litter type across different chemotypes from the same plant species. This result suggests an important role of plant chemical defense on microbial community composition. However, patterns of diversity and potential links to microbial function were inconsistent. This inconsistency occurred because the ranking of different microbial taxa across chemotypes did not correspond to our understanding of function and neither soil nor litter source blocked together when we sorted treatments by the relative abundance of individual or functionally similar taxa. The significance of the changes in microbial community composition will therefore likely require an analysis of functional outcomes (i.e., nutrient cycling, enzyme activity) to be understood.

## Data Availability

Sequence reads were deposited in the NCBI SRA database and are available under the BioProject ID PRJNA487727. https://www.ncbi.nlm.nih.gov/bioproject/?term=PRJNA487727.
